# Spirituality in people with advanced chronic obstructive pulmonary disease – challenge for more effective interventions, support, and healthcare education: Mini-review

**DOI:** 10.3389/fmed.2022.954519

**Published:** 2022-12-06

**Authors:** Aleksandra Kotlińska-Lemieszek, Małgorzata Fopka-Kowalczyk, Małgorzata Krajnik

**Affiliations:** ^1^Pharmacotherapy in Palliative Care Laboratory, Chair and Department of Palliative Medicine, Poznań University of Medical Sciences, Poznań, Poland; ^2^Outpatient Palliative Medicine Clinic, Heliodor Swięcicki University Hospital, Poznań, Poland; ^3^Department of Philosophy and Social Sciences, Nicolaus Copernicus University in Toruń, Toruń, Poland; ^4^Department of Palliative Care, Collegium Medicum in Bydgoszcz, Nicolaus Copernicus University in Toruń, Bydgoszcz, Poland

**Keywords:** spirituality, religiosity, spiritual well-being, religious/spiritual coping, interventions, chronic obstructive pulmonary disease, COPD

## Abstract

More recently there has been a growing interest in spirituality in medicine, especially in the field of palliative care, oncology, intensive care, and cardiology. However, according to literature, it seems to be a limited number of researches on how healthcare professionals should provide spiritual care (SC) for people with non-malignant lung diseases and what kind of education for them enables them to do it efficiently. This mini-review aims to provide an overview of current knowledge of an area of spirituality and SC for people with advanced chronic obstructive pulmonary disease, including spiritual well-being and religious/spiritual coping, their relations with the quality of life and symptom burden, exercise capacity and daily functioning, mental health, or medication adherence. It also analyses the use of interventions to meet patients’ spiritual needs and patients’ expectations regarding SC provided by professional careers. Based on the literature authors try to show the fields that should be improved and proposed future research directions.

## Introduction

One of the dimensions of growing significance in modern medicine is a holistic approach to patient care with the recognition of the central place of spirituality in a person’s life (1, 2).

According to European Association for Palliative Care (EAPC) and Polish Association for Spiritual Care in Medicine (PASCiM), spirituality is multidimensional and includes religiousness of a person, existential quests, and value-based considerations (3–5). The evidence shows a favorable impact of higher level of religiosity/spirituality or greater spiritual well-being (SWB) on survival (6), coping with disease (7), patient’s satisfaction with treatment and care (8), less depression (9), lower anxiety (10), or better resilience (11). Thus, besides palliative care, spiritual care (SC) has started to be recognized as an integral part of care especially for people with cancer (12), heart failure (13), or admitted to an intensive care unit (14). Much less is known about spirituality in patients with advanced chronic lung diseases even though they usually suffer from breathlessness, cough, fatigue, fears of suffocation, numerous social limitations, anxiety, and depression every day (15, 16).

The purpose of this mini-review is to provide a comprehensive analysis of the current knowledge on the role of spirituality in people with advanced chronic obstructive pulmonary disease (COPD) and to propose future research directions and potential role of SC for these patients.

### Spiritual well-being of individuals with COPD

Spiritual well-being of individuals with COPD has been evaluated in a number of studies (17–24) (Table 1). It was found to be similarly low among people with COPD and with inoperable lung cancer (18). SWB was higher in patients with mild airflow limitation, experiencing fewer COPD exacerbations and black individuals, and lower in those with more symptom burden, physical impairment and poor mental health as well as current smokers (18, 20, 21, 24). Also patients with advanced pulmonary diseases (50% with COPD) on a waiting list for a lung transplantation presented a higher SWB as compared to patients not considered for a lung transplant (22). In COPD patients with a moderate level of SWB, the religious component was shown to have more significant contribution than the existential one (17). In a longitudinal study, SWB remained relatively stable over time (median 15 months) in people with advanced congestive heart failure (CHF) and COPD (18).

**TABLE 1:** Benefits and impacts of spirituality among patients with COPD according to the literature.

Main findings
***Spiritual well-being*** SWB is comparably low in people with advanced COPD and inoperable lung cancer (20). SWB is lower in COPD patients with higher symptom burden, poor mental state, with more disease exacerbations and in current smokers (18, 20, 21, 24). SWB is higher in individuals waiting for a lung transplantation (22).
***Religious/spiritual coping in disease*** COPD patients mostly use positive RC, such as seeking God’s love and care and asking forgiveness for sins. They, however, employ negative RC, such as questioning God’s love and punishing God reappraisal, more often than healthy individuals (19, 24–28).
***Spiritual well-being and quality of life*** High sense of spirituality correlates with lower stress and higher QoL (34). Higher negative RC correlate with worse QoL, while positive RC are associated with a better QoL (19, 24, 29).
***Spiritual well-being and symptom burden*** SWB negatively correlates with symptom distress/burden (18, 21, 24) Patients who present a higher level spirituality and SWB may experience less dyspnea, fewer symptoms of anxiety and depression (20, 24, 35). Individuals who utilize more negative RC show more dyspnea, anxiety and depressive symptoms (24, 26, 27).
***Spirituality, exercise capacity, daily functioning and pulmonary function*** Patients with higher SWB show better resilience, self-management and medication adherence (21, 23)
***Interventions to improve spiritual well-being*** At hospital daily visits of chaplains may decrease anxiety, shorten length of hospital stay and increase satisfaction with quality of care (40). At home clergy-laity support is related to the benefit to patients’ mental health (41). Pulmonary rehabilitation (PR) promotes improvement in anxiety, depression, dyspnea, exercise capacity and QoL. It increases ORA, positive RC and decreases negative RC (37). Dignity Therapy may have positive impact on well-being of patients with advanced COPD with a short life prognosis. It may help them to identify and meet their spiritual needs (42)
***Patients’ expectations regarding spiritual care provided by professional carers*** Almost all and almost half of patients who declare to have or not to have religious and spiritual beliefs, respectively, agree that physicians should ask patients about their beliefs when they become gravely ill (43). Physicians rarely discuss spiritual/religious issues with their patients even at their end-of-life (47). Two thirds of patients state that their trust in a physician would increase if he/she asked them about spiritual matters (43)

QOL, quality of life; SWB, spiritual well-being; ORA, organizational religiosity; RC, religious coping.

### Religious/spiritual coping in patients with COPD

Chronic obstructive pulmonary disease patients mostly use positive religious coping (RC), such as seeking God’s love and care, looking for control through a partnership with God, benevolent religious reappraisal, and asking forgiveness for sins (19, 24–28). However, they also use negative RC such as questioning God’s love and punishing God’s reappraisal more often than healthy individuals (19, 25, 26). Some factors such as sex or nationality and culture are important. Women were shown to present a higher positive RC than men (27). Dutch patients who reported at least a little faith in God or a spiritual power employ positive RC more often than non-believers (19). However, Brazilians applied more positive and less negative RC as compared to Dutch patients (29). Praying, support within the religious community, church attendence, sign of the cross, and icons of saints were helpful in coping with the burden of the disease among the Greek (30) and more than half of Polish COPD patients (31). Also religious/spiritual ceremonies give patients some hope and a sense of meaning while dealing with an illness. Those who are regular churchgoers ask for God’s help and try to find spiritual support and a church is a safe place for them (30).

More than a half of Polish patients believed that God had a plan for their lives and would not allow illness without a reason (31). Some patients with COPD experience also guilt because of smoking before illness and to deal with it use different strategies such as active or passive acceptance (32). Some had to face helplessness (33). Both emotions exert the impact on patients’ daily life and coping with an illness. Self-blame appeared to intensify feelings of helplessness and passive resignation, as well as poor self-management (33). For patients who focused on faith in God, church and family provided a more positive effect and existed alongside helplessness. They did not experience self-blame but they articulated strongly held beliefs in God, the Church and family and repeatedly reported those to be the most important things that helped them live with their illness (33).

### Spiritual well-being and quality of life of COPD patients

A number of studies demonstrated positive associations between SWB and quality of life (QoL) and more varied outcomes relating to associations between religiosity and QoL in COPD patients (17–19, 22, 24, 29, 34). A high sense of spirituality was shown to correlate with lower stress and higher QoL (34). Total SWB measured by FACIT-Sp-12 was positively associated with emotional function and mastery evaluated by Chronic Respiratory Disease Questionnaire (CRQ), while two SWB domains: meaning and peace with total scores of CRQ (24). Faith domain of SWB of FACIT-Sp-12 and religiosity measured with a Duke Religion Index (DUREL) was not associated with QoL in this study (24). Similar results were reported by other authors (22), while according to others, faith subscale of FACIT-Sp score positively correlated with the scores of Multidimensional Index of Life Quality (MILQ) (18). Also increased religiosity measured with the three components of DUREL (organizational religiosity, ORA; non-organizational religiosity, NORA; and intrinsic religiosity, IR) was associated with a better QoL of COPD patients (29). Individuals having at least a little faith in God had a higher QoL compared to individuals with beliefs in a spiritual power only (19). Individuals with higher SWB as well as religious well-being reported higher satisfaction with the treatment, one of health-related QOL domains measured using the Seattle Obstructive Lung Disease Questionnaire (SOLDQ) (17). QoL of COPD patients was also shown to be associated with strategies of coping with the disease. Higher negative RC was correlated with worse QoL (19, 24, 29), while positive RC associated with a better QoL of COPD patients (29). Furthermore, positive RC positively and negative RC inversely correlated with patients’ satisfaction with life (28).

### Spiritual well-being and symptom burden in COPD patients

The relationship between spirituality and RC and symptom burden in COPD patients were investigated in a number of publications (18, 20–22, 24–27, 34). SWB and its “peace” domain measured using FACIT-Sp-12 were demonstrated to be negatively correlated with symptom distress/burden measured using the Memorial Symptom Assessment Scale-Global Distress or the COPD Assessment Test (CAT) (18, 21, 24). Although not all (34), some studies show a negative association between SWB and religiosity and dyspnea (20, 24, 35). Patients who experience more breathlessness assessed by the modified Medical Research Council (mMRC ≥ 2) presented lower levels of SWB (20). A higher score on the “peace” domain of FACIT-Sp-12 was negatively associated with breathlessness (total, affective, and physical components) measured by Dyspnoea-12. The total score of FACIT-Sp-12 was inversely correlated with the affective component of dyspnea. A higher level of the total and affective component of dyspnea was also demonstrated in individuals presenting more negative RC (24).

Higher SWB was also associated with less anxiety and depression in COPD individuals (24, 36). Total scores of FACIT-Sp-12 as well as “peace” and “meaning” domains were negatively correlated with anxiety (24). Moreover, total scores and scores for all three domains of FACIT-Sp-12 (“peace,” “meaning,” and “faith”) as well as IR were negatively associated with depression (24). Also, individuals who utilize more negative RC showed more anxiety (24) and depressive symptoms (24, 26, 27). Interpreting the lung disease as punishment from God was the strongest predictor of trait anxiety, depression, and psychosocial disability (25).

Spirituality and faith have been shown to prevent depression, suicidal thoughts, and improve hope and dignity (15). Religious beliefs and spiritual activities were associated with less severe illness and fewer prior psychiatric problems (35). People with CHF and chronic pulmonary disease who prayed or studied the Bible daily or more often were less likely to report prior psychiatric problems (35).

### Spirituality, exercise capacity, daily functioning, and pulmonary function of COPD patients

Associations between levels of spiritual and religious well-being, RC and physical capacity of COPD patients have so far been understudied (17, 18, 22, 24, 27, 29, 37). Religious activities and intrinsic religious attitudes were inversely related to the severity of the medical illness, physical disability, or perceived shortness of breath, while religious activities (especially religious attendance) were associated with greater social support (35). Physical impairment measured with the Sickness Impact Profile (SIP) in patients with COPD and CHF negatively correlated with SWB (18). A higher score on “peace” domain of FACIT-Sp-12 was found to be associated with a better exercise capacity measured using the 6-min walk test (6MWT) in individuals waiting for lung transplantation (22). The distance covered in 6MWT was strongly inversely associated with negative RC in individuals with moderate and severe COPD (27). Other publications presented negative or no associations between spirituality/religiosity and patients’ functioning (17, 24, 29). For example, Silva et al. found negative correlation between religious well-being assessed as a component of SWB and physical functioning in a sample of COPD patients with severely compromised physical function (17). Of note, no association between physical function and the total score of SWB and the score of its existential component was found in this study. Also, increased religiosity and increased positive RC were shown to be associated with shorter distance covered (6MWT) in COPD patients from Brazil and the Netherlands (29).

Importantly, in a study by Chen et al., the authors found positive correlations between SWB and resilience as well as four dimensions of patients’ self-management (symptom management, daily life management, emotion management, and self-efficacy) (23). COPD patients with higher SWB showed better medication adherence (21).

Only a few studies explored relationships between spirituality and pulmonary function with varied outcomes (21, 22, 29). For example, SWB was positively associated with FEV1/FVC values (21) and NORA (DUREL) was negatively associated with forced expiratory volume in the first second (FEV1) (29). The latter result seems to reflect the fact that patients with more advanced disease turn to religion and spirituality.

### Interventions to improve spiritual well-being of patients with COPD

Spiritual care is provided by healthcare providers (basic SC) who address spiritual concerns and cooperate with trained chaplains (specialist SC) who especially deal with unmet spiritual needs or spiritual distress of the patients. Apart from the referral to a chaplain as the best proven spiritual intervention, several others focused on finding a meaning of life, supporting dignity, or helping to review own life have been shown to be effective, especially for people at their end-of-life (38, 39).

However, the literature search indicated very few studies regarding interventions aiming at improving specifically COPD patients’ spiritual functioning. Daily visits from chaplains in patients with COPD resulted in a significant decrease in anxiety compared to the non-visited controls and to a shorter length of hospital stay and satisfaction with the quality of care (40). In the case of home care, visits from clergy or other members of a patient religious community were significantly and inversely related to depression among seriously ill patients including people with advanced COPD (41). Patients who were visited often by clergy and laity reported significantly fewer symptoms of depression than those visited only a little.

Following pulmonary rehabilitation increase in positive RC and ORA, and a decrease in negative RC was observed in a non-randomized controlled trial (37). Pulmonary rehabilitation promoted improvement in anxiety, depression and depressive symptom severity, exercise capacity, dyspnea, and QoL. As already mentioned, changes in ORA and positive RC positively correlated with increases in patients’ exercise capacity (measured as 6MWT) and negatively with CAT, which reflected better QoL (37).

Dignity therapy (DT) has been shown to have a positive impact on the well-being of the patients with advanced COPD with a short life prognosis (42). Such interventions well received by patients may help them in recognizing and fulfilling their spiritual needs in the last phase of their life.

### COPD patients’ expectations regarding spiritual care provided by professional carers

Studies show that patients with chronic diseases wish to talk to doctors about spiritual issues. This may, however, vary in different populations (19, 43–45). Almost all (94%) ambulatory patients visiting a pulmonary faculty office (no specific diagnoses presented) who declared to have religious and spiritual beliefs and 45% of those not declaring religious and spiritual beliefs agreed that physicians should ask patients about their beliefs if they become gravely ill (43). Approximately 45% percent of participants declared religious or spiritual beliefs that would influence their medical decisions in a severe disease. Approximately 66% stated that their trust in a physician would increase if he/she asked about spiritual matters (43). However, among newly diagnosed individuals with severe chronic lung diseases none of the participants wanted to discuss religious and spiritual issues with a professional and few recalled to have had such a need in the past (19).

Asking about spiritual and religious beliefs was one of four issues which physicians rarely discussed during communication about end-of-life care. Approximately 82.6% of oxygen-dependent COPD patients reported not having been asked about religious and spiritual beliefs (46). In a survey among Polish pulmonologists, about 16% respondents talk on spiritual/religious needs to their patients with advanced COPD routinely (always or often) contrary to almost 29% never discussing those issues (47).

## Discussion

**FIGURE 1:**
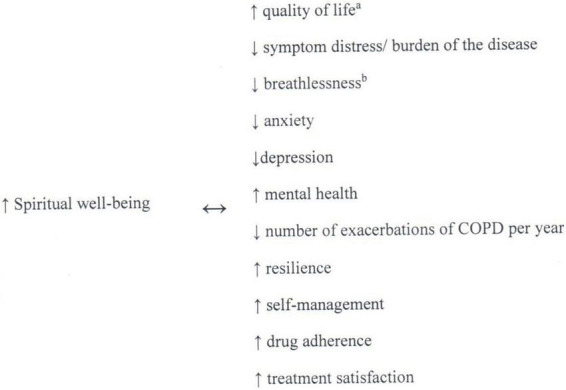
Associations between spiritual well-being and clinical outcomes (17–24, 29, 34). Non-casual-relationships can be proved on the base of studies identified. ^a^no association in patients with COPD in a study by Hasegawa et al. (20). ^b^no association in a study by Delgado et al. (34).

This mini-review provides an analysis of the important role of SWB and RC of people with advanced COPD. It also discusses what has been proven, and what still needs to be researched, on the relations between SWB and QoL, symptom burden, resilience, self-management, satisfaction with the treatment and with own life, physical functioning and mental health, or medication adherence (Figure 1).

Unfortunately, despite increasing evidence on the importance of patient spirituality, still very little is known about the efficacy of SC and the interventions directed to meet the patient spiritual needs. Much more has been proven in many other fields of medicine. For example, in intensive care, the chaplain activities (48) have been examined in detail including evidence of higher family members’ satisfaction with SC if a pastor or spiritual advisor was involved within 24 h of patient death (49), or the association of chaplaincy services with significantly lower rates of hospital intensive care units deaths and higher rates of hospice enrollment (50). In case of hospitalized COPD patients, daily visits of chaplains exerted a positive impact on anxiety, length of hospital stay, and satisfaction with the quality of care (40). However, as these patients usually spend much more time suffering from symptom burden and many limitations not in hospital but at their homes, the preliminary observation that clergy-laity support is related to the benefit to their mental health, gives an argument for involving SC into integrated care for people with COPD (41). Taking into account, the widely accepted recommendation on pulmonary rehabilitation for COPD patients, its potential influence on ORA and positive RC is very promising and should be further studied (37). Besides the preliminary observations on the role of chaplaincy, pulmonary rehabilitation, and DT; the latter shown to be helpful in fulfilling the spiritual needs of patients with COPD at the end of their life (42), there is a lack of research on other interventions. The recommendation of respiratory societies could help to change this situation, as it seems to be the case in other branches of medicine. Taking into account the negative impact of the spiritual crisis on patients coping with cancer, its physical symptoms, and its treatment, National Comprehensive Cancer Network presented to clinicians some standards of care, based on multidisciplinary team and cooperation with a certified chaplain (12). American Society of Clinical Oncology gave a strong recommendation for clinicians to explore how a patient’s culture, religion, or spiritual belief system affects their end-of-life decision making or care preferences along with the strategies on communication, using standardized tool to assess a patient’s spiritual or religious beliefs and in case of spiritual distress – cooperation with a medically trained chaplain (51). In case of the adult cancer patient at the end of life, European Society for Medical Oncology recommended routine assessment of spiritual distress, using compassionate listening, some specifically proven interventions and referral to a trained chaplain or SC professional (38). American College of Critical Care Medicine even recommended that healthcare provider pray with the patient who requested it if a clinician felt comfortable with it, as a part of holistic intensive care (52). Similar guidelines, as mentioned above, published by respiratory societies could promote more efficient approach for clinicians to the spiritual needs of people with advanced COPD. Our mini-review also revealed the great need of improving the education of healthcare professionals on SC. On the one hand, many COPD patients wish to talk to doctors about spiritual issues (43). On the other hand, a huge minority of doctors do it as a routine approach (47). However, education how to provide SC especially for people at the end of life is not only about communication, using diagnostic tools or implementing specific interventions, which, by the way, are mandatory components of SC. According to the European Association for Palliative Care, the first recommendation for the training of clinicians caring for people at the end of life is the development of the reflective capacity of staff to consider the importance of spiritual dimensions in their own lives (3). Only by learning how to care about ourselves, will we be able to care for other people including supporting them spiritually.

## Conclusion and future directions

Spirituality and SWB of people with advanced COPD are related with their QoL, symptom burden, resilience, self-management, satisfaction with the treatment and with their own life, physical functioning and mental health, or medication adherence. However, there are some emerging challenges such as proving the efficacy of interventions aimed at meeting patients’ spiritual needs, preparation of respiratory societies’ guidelines on SC, and the implementation of optimal education for healthcare professionals caring for people with advanced COPD.

## Author contributions

AK-L developed the concept and structure and wrote the first draft of the manuscript. MF-K and MK contributed to the writing of the manuscript. All authors reviewed and approved the final manuscript.
